# A case report of pulmonary *Botrytis* sp. infection in an apparently healthy individual

**DOI:** 10.1186/s12879-019-4319-2

**Published:** 2019-08-02

**Authors:** Seishu Hashimoto, Eisaku Tanaka, Masakuni Ueyama, Satoru Terada, Takashi Inao, Yusuke Kaji, Takehiro Yasuda, Takashi Hajiro, Tatsuo Nakagawa, Satoshi Noma, Gen Honjo, Yoichiro Kobashi, Noriyuki Abe, Katsuhiko Kamei, Yoshio Taguchi

**Affiliations:** 1 Department of Respiratory Medicine, Tenri Hospital, 200 Mishima-cho, Tenri, Nara, 632-8552 Japan; 2 Department of Thoracic Surgery, Tenri Hospital, 200 Mishima-cho, Tenri, Nara, 632-8552 Japan; 3 Department of Radiology, Tenri Hospital, 200 Mishima-cho, Tenri, Nara, 632-8552 Japan; 4 Department of Pathology, Tenri Hospital, 200 Mishima-cho, Tenri, Nara, 632-8552 Japan; 5 Department of Clinical Laboratory, Tenri Hospital, 200 Mishima-cho, Tenri, Nara, 632-8552 Japan; 6 Division of Clinical Research, Medical Mycology Research Center, Chiba University, 1-8-1 Inohana, Chuo-ku, Chiba, 260-8673 Japan

**Keywords:** *Botrytis* sp., Pulmonary infection, Immunocompetent host, DNA sequence analysis

## Abstract

**Background:**

*Botrytis* species are well known fungal pathogens of various plants but have not been reported as human pathogens, except as allergenic precipitants of asthma and hypersensitivity pneumonitis.

**Case presentation:**

The asymptomatic patient was referred because of a nodule revealed by chest X-ray. Computed tomography (CT) showed a cavitary nodule in the right upper lobe of the lung. He underwent wedge resection of the nodule, which revealed necrotizing granulomas and a fungus ball containing Y-shaped filamentous fungi, which was confirmed histopathologically. Culture of the specimen yielded white to grayish cotton-like colonies with black sclerotia. We performed multilocus gene sequence analyses including three single-copy nuclear DNA genes encoding glyceraldehyde-3-phosphate dehydrogenase, heat-shock protein 60, and DNA-dependent RNA polymerase subunit II. The analyses revealed that the isolate was most similar to *Botrytis elliptica*. To date, the pulmonary *Botrytis* sp. infection has not recurred after lung resection and the patient did not require any additional medication.

**Conclusions:**

We report the first case of an immunocompetent patient with pulmonary *Botrytis* sp. infection, which has not recurred after lung resection without any additional medication. Precise evaluation is necessary for the diagnosis of pulmonary *Botrytis* infection because it is indistinguishable from other filamentous fungi both radiologically and by histopathology. The etiology and pathophysiology of pulmonary *Botrytis* infection remains unclear. Further accumulation and analysis of *Botrytis* cases is warranted.

**Electronic supplementary material:**

The online version of this article (10.1186/s12879-019-4319-2) contains supplementary material, which is available to authorized users.

## Background

*Botrytis* species are important pathogens of nursery plants, vegetables, orchard crops, and can colonize stored and transported agricultural products [1]. In particular, *Botrytis cinerea* is responsible for gray mold disease on more than 200 host plants [2] and has been isolated from numerous places around the world, especially humid, temperate and subtropical regions [1–3]. In contrast, winegrowers and viniculturists sometimes welcome *B. cinerea*, which facilitates a concentrated sweet wine in the right conditions. With regard to other *Botrytis* species, *B. squamosa*, *B. allii*, and *B. aclada* attack bulbs of onion, garlic and leek, while *B. tulipae* and *B. elliptica* attack flower bulbs such as tulip and lily.

Airborne exposure to *Botrytis* sp. has been reported globally but the prevalence of *Botrytis* is different by regions and seasons. *Botrytis* exposure is especially significant in occupational setting such as greenhouses and grain mills. Although many people may inhale spores of *Botrytis* species, it is of interest that *Botrytis* species have not been reported as human pathogens, except as allergenic precipitants of asthma and hypersensitivity pneumonitis [3].

We report an apparently immunocompetent Japanese man with pulmonary *Botrytis* sp. infection, which to date has not recurred after lung resection.

Preliminary results of this case report have been presented in a poster discussion at the annual meeting of the American Thoracic Society [4].

## Case presentation

A 62-year-old Japanese man from Tenri City in Nara prefecture was referred to our hospital because of a nodular shadow on chest X-ray taken at a regular health checkup. He did not have cough, sputum, hemoptysis, fever, night sweats, chest pain and weight loss. He had smoked two packs of cigarettes per day for 30 years until 12 years previously and occasionally consumed alcohol. He was an office worker and had been to China and Taiwan on business for several days approximately 30 years previously. He had no known occupational or inhalational exposures. He had never grown any plants, fruits, or vegetables. He had diabetes mellitus, hyperlipidemia and gout and had been treated with a combination of mitiglinide and voglibose, vildagliptin, rosuvastatin, and allopurinol. He had no known tuberculosis, bronchiectasis, or allergies. Family history was unremarkable and there was no family history of fungal disease. On examination, his temperature was 36.4 °C, blood pressure 144/88 mmHg, and resting pulse was 85 beats per minute, with 12 breaths per minute. The oxygen saturation was 98% while breathing ambient air. He had normal vesicular sounds on auscultation. The remainder of the examination was normal.

**Fig. 1:**
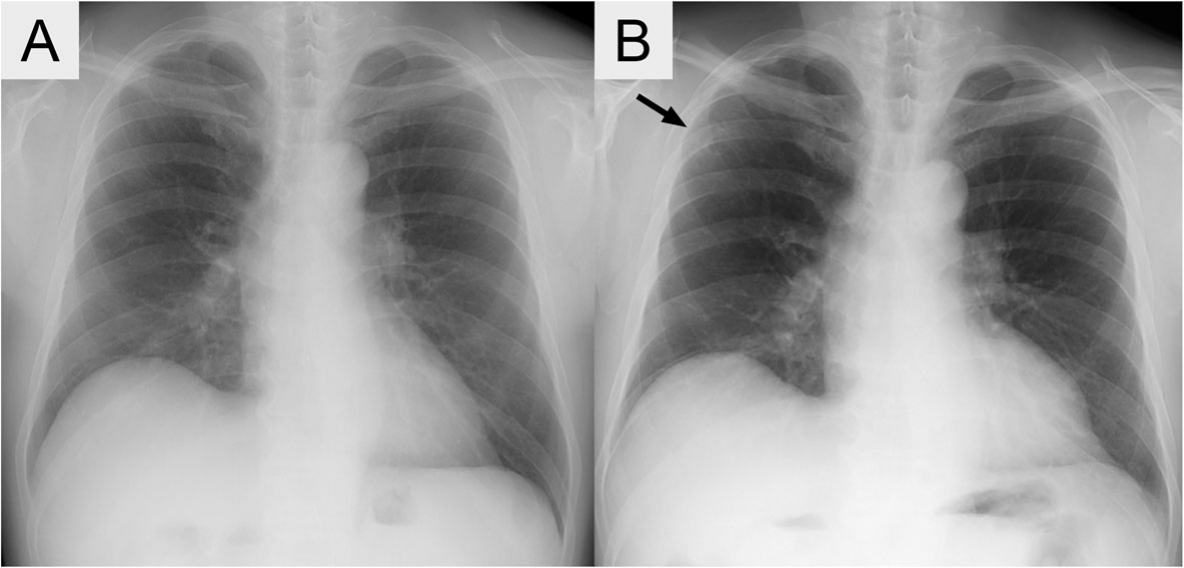
Imaging studies of the chest. **a** A chest X-radiograph obtained 18 months earlier reveals no abnormal findings. **b** A chest X-radiograph on admission shows a cavitary nodule (arrow) in the right upper lung field.

**Fig. 2:**
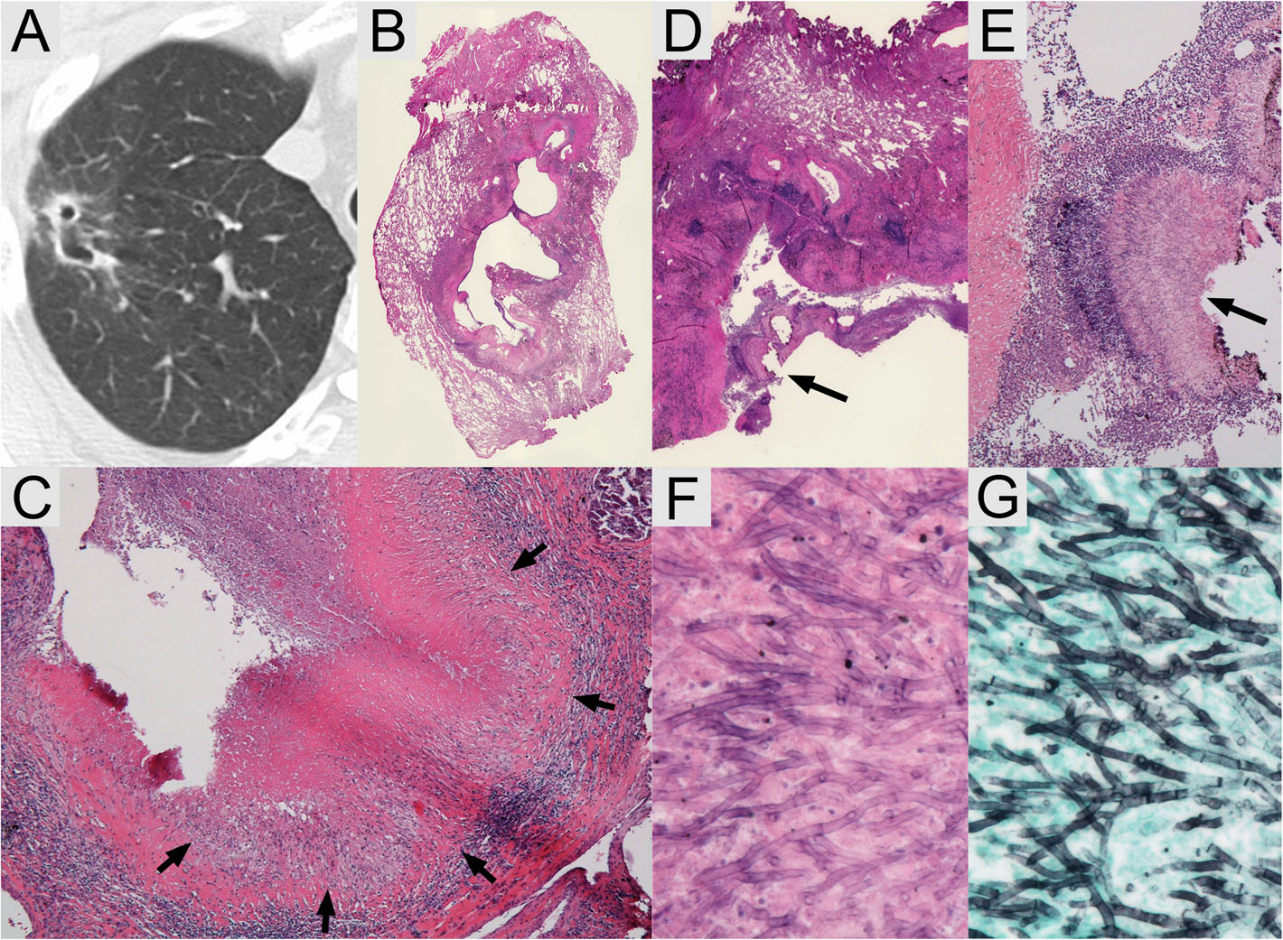
Imaging study of the chest and histopathological images of resected specimens. **a** Computed tomography of the chest reveals a cavitary nodule with thin wall and pleural indentations in the right upper lobe. **b** The bronchioles surrounding the areas of inflammation and fibrosis are seen in hematoxylin and eosin stained section through a magnifying glass. **c** The necrotizing granuloma (arrows) destroying and invading the wall of bronchiole is seen in hematoxylin and eosin stained section at low magnification. **d** A fungus ball (arrow) is seen in the cavity at the other hematoxylin and eosin stained section through a magnifying glass. **e** A fungus ball (arrow) is shown at low magnification view in hematoxylin and eosin stained section. **f** Y-shaped filamentous fungi in the fungus ball are seen in hematoxylin and eosin stained section at higher magnification. **g** The organisms are well outlined with Grocott’s methenamine silver stain. The dichotomous branching hyphae and scatter septations can be observed at higher magnification

A chest X-radiograph showed a cavitary nodule in the right upper lung field (Fig. 1b), which was not seen on X-ray film taken 18 months previously (Fig. 1a). Computed tomography (CT) revealed a nodule with cavitary lesion, measuring 25 mm in diameter, in the right upper lobe (Fig. 2a). ^18^F-fluorodeoxy-glucose positron emission tomography revealed mild accumulation in the nodule (maximum standardized uptake value = 2.2). Laboratory tests (Table 1) showed that serum creatinine was elevated at 1.2 mg/dL. The hemoglobin level, white cell and platelet counts, and results of coagulation and liver function were normal. C-reactive protein was below 0.2 mg/dL. The erythrocyte sedimentation rate was slightly elevated at 12 mm/h. Serum tests for 1, 3-β-D-glucan, *Aspergillus* antigen, anti-*Aspergillus* antibody, and *Cryptococcus* antigen were all negative. Hemoglobin A1c was normal at 5.6%. Both anti-human immunodeficiency virus antibody and anti-human T-cell leukemia virus type 1 antibody were negative. Bronchoscopic examination was performed, and bronchial brush specimens and bronchial washing fluids were obtained under fluoroscopy. However, the examination revealed no remarkable malignant cells or microorganisms including fungi and mycobacteria. Wedge resection of the cavitary nodule in the right upper lobe using video-assisted thoracoscopy was undergone to diagnose the nodule and exclude malignancy.

**Table 1:** Laboratory data

Variable	On Admission	Reference range, Adults
Hemoglobin (g/dl)	14.1	13.1–17.0
Hematocrit (%)	39.3	38.0–50.0
White cell count (per mm^3^)	6820	3500–8500
Differential count (%)		
Neutrophils	72	46–73
Lymphocytes	23	20–45
Monocytes	4	1–7
Eosinophils	1	1–3
Platelet count (per mm^3^)	188,000	150,000-350,000
Erythrocyte sedimentation rate (mm/hour)	12	1–10
Blood urea nitrogen (mg/dl)	16	7–19
Creatinine (mg/dl)	1.2	0.6–1.2
Total protein (g/dl)	7.3	6.7–8.1
Albumin (g/dl)	4.4	4.0–5.0
Lactate dehydrogenase (U/liter)	213	100–225
Asparate aminotransferase (U/liter)	20	11–32
Alanine aminotransferase (U/liter)	18	3–30
Total bilirubin (mg/dl)	0.8	0.2–1.0
γ-Glutamyltransferase (U/liter)	28	10–60
Alkaline phosphatase (U/liter)	241	100–335
C-reactive protein (mg/dl)	< 0.2	< 0.2
Sodium (mmol/liter)	142	139–147
Potassium (mmol/liter)	4.1	3.5–4.8
Chloride (mmol/liter)	107	101–111
Blood sugar (mg/dl)	123	65–110
Prothrombin time (International normalized ratio)	1.00	0.86–1.12
Activated partial thromboplastin time (second)	27.3	23.0–35.0
Hemoglobin A1c (%)	5.8	4.3–6.1
Carcinoembryonic antigen (ng/ml)	2.8	< 5.0
Cytokeratin 19 fragment (ng/ml)	2.7	< 3.5
Progastrin-releasing peptide (pg/ml)	84.9	< 81
Soluble interleukin-2 receptor (U/ml)	434	145–519
1, 3-β-D-glucan (pg/dl)	< 5.5	< 11
*Aspergillus* antigen (cut off index)	< 0.1	< 0.5
Anti-*Aspergillus* antibody	Negative	Negative
*Cryptococcus* antigen	Negative	Negative
Anti-human immunodeficiency virus antibody (cut off index)	0.16	< 1.0
Anti-human T-cell leukemia virus type 1 antibody (cut off index)	0.09	< 1.0

Histopathological examination of the resected specimen showed necrotizing granulomas and a fungus ball containing Y-shaped filamentous fungi (Fig. 2b-f). Grocott’s methenamine silver stain revealed that the septated hyphae branched dichotomously (Fig. 2g). Vascular invasion was not seen in the tissue. The histopathological features resembled those of chronic cavitary pulmonary aspergillosis.

**Fig. 3:**
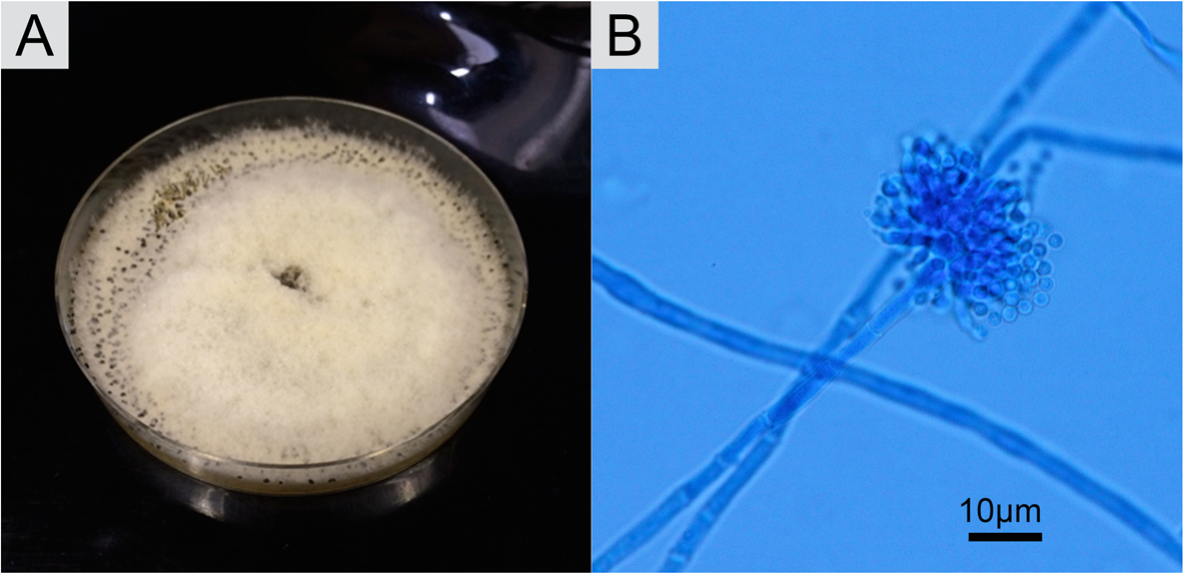
Morphological characters of the colony. **a** The white to grayish cotton-like colonies with black sclerotia were cultivated from lung biopsy material on potato dextrose agar for 7 days at 25 °C. **b** The mycelium and conidiophores were septated, and the conidia were egg-shaped hyaline

**Fig. 4:**
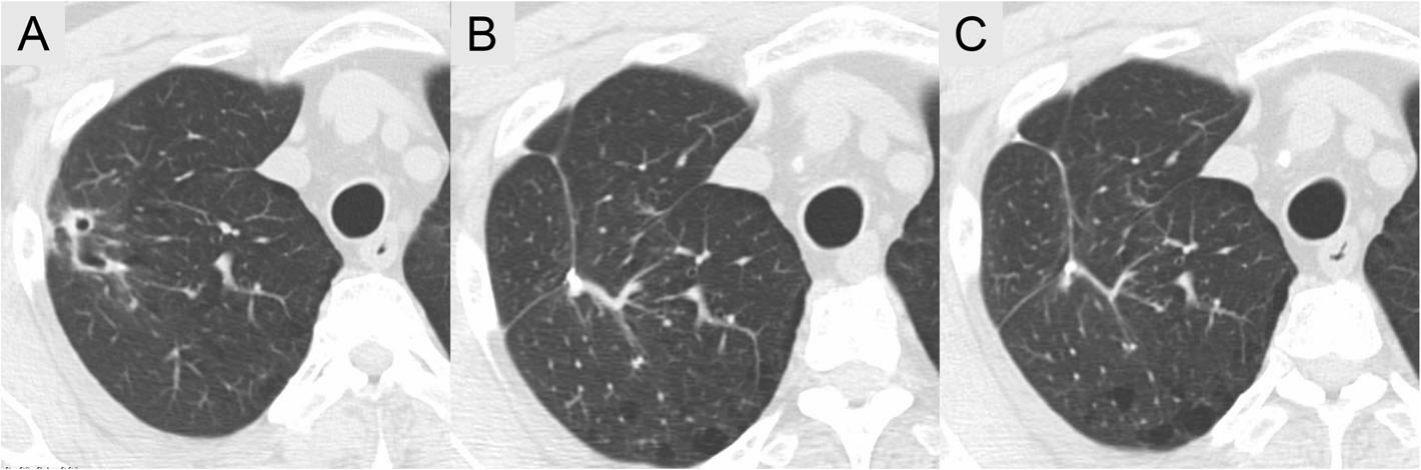
Subsequent CT scans of the chest. **a** Computed tomography (CT) of the chest on admission reveals a cavitary nodule with thin wall and pleural indentations in the right upper lobe. **b** CT of the chest obtained 1 year after surgery shows an operation scar without any nodules or cavities. **c** CT of the chest obtained 3 years after surgery shows the same findings as CT 1 year after surgery (**b**)

The lung biopsy material was cultured for fungi, mycobacteria and other bacteria. Because molds were observed in the intraoperative frozen section, culture for fungi was incubated at 25 °C on potato dextrose agar (PDA) for 7 days. The culture yielded white to grayish cotton-like colonies with black sclerotia around the colonies (Fig. 3a). No other microorganisms were isolated. The multilocus gene sequence analyses identified the fungus as *Botrytis* sp. as shown in Tables 2, 3, 4 and Additional file 1: Figures S1-S5. He was diagnosed with pulmonary *Botrytis* sp. infection. As the patient was immunocompetent and the completely resected specimen showed necrotizing granulomas without vascular invasion, he was not offered antifungal agents. The pulmonary *Botrytis* sp. infection has not recurred 3 years after resectional surgery (Fig. 4).

**Table 2:** List of primers employed in polymerase chain reaction in this study

Target Region	Primer Name	Primer Sequence (5’ to 3’)	Reference
ITS	ITS1	TCCGTAGGTGAACCTGCGG	[4]
	ITS4	TCCTCCGCTTATTGATATGC	
Domain 1 and 2	NL-1	GCATATCAATAAGCGGAGGAAAAG	[5]
	NL-4	GGTCCGTGTTTCAAGACGG	
G3PDH	G3PDHfor	ATTGACATCGTCGCTGTCAACGA	[6]
	G3PDHrev	ACCCCACTCGTTGTCGTACCA	
HSP60	HSP60for	CAACAATTGAGATTTGCCCACAAG	[6]
	HSP60rev	GATGGATCCAGTGGTACCGAGCAT	
RPB2	RPB2for	GATGATCGTGATCATTTCGG	[6]
	RPB2rev	CCCATAGCTTGCTTACCCAT

**Table 3:** BLAST results of sequences at internal transcribed spacer (ITS) region and domain 1 and 2 region

	ITSfor	ITSrev	Domain 1 and 2for	Domain 1 and 2rev
1	*Botryotinia fuckeliana*	*Botrytis cinerea*	*Botrytis cinerea*	*Botrytis cinerea*
Identities	489/491 (99%)	482/483 (99%)	559/560 (99%)	558/559 (99%)
Accession no.	KF533033.1	MF521935.1	KU140653.1	KU729179.1
2	*Botrytis fabiopsis*	*Botrytis cinerea*	*Botrytis cinerea*	*Botrytis cinerea*
Identities	489/491 (99%)	482/483 (99%)	559/560 (99%)	558/559 (99%)
Accession no.	KR135152.1	KU291996.1	KR094468.1	KT323330.1
3	*Botrytis elliptica*	*Botrytis cinerea*	*Botrytis cinerea*	*Botrytis cinerea*
Identities	489/491 (99%)	482/483 (99%)	559/560 (99%)	558/559 (99%)
Accession no.	KR055047.1	KT271762.1	KP671724.1	KR094468.1
4	*Botrytis cinerea*	*Botrytis elliptica*	*Botrytis cinerea*	*Botrytis cinerea*
Identities	489/491 (99%)	483/484 (99%)	559/560 (99%)	558/559 (99%)
Accession no.	KR002909.1	KR055047.1	CP009808.1	KP780471.1
5	*Botrytis cinerea*	*Botrytis elliptica*	*Botrytis cinerea*	*Botrytis cinerea*
Identities	489/491 (99%)	483/484 (99%)	559/560 (99%)	558/559 (99%)
Accession no.	KF859919.1	KR076789.1	KM249092.1	KP671724.1

**Table 4:** BLAST results of sequences at glyceraldehyde-3-phosphate dehydrogenase (*G3PDH*), heat-shock protein 60 (*HSP60*) and DNA-dependent RNA polymerase subunit II (*RPB2*) genes

	G3PDHfor	G3PDHrev	HSP60for	HSP60rev	RPB2for	RPB2rev
1	*B. elliptica*	*B. elliptica*	*B. elliptica*	*B. elliptica*	*B. elliptica*	*B. elliptica*
Identities	924/928 (99%)	924/929 (99%)	988/988 (100%)	981/983 (99%)	961/963 (99%)	992/993 (99%)
Accession no.	KP896523.1	KP896523.1	KR076786.1	KR076786.1	KR076787.1	KR076787.1
2	*B. squamosa*	*B. squamosa*	*B. squamosa*	*B. squamosa*	*B. elliptica*	*B. elliptica*
Identities	880/884 (99%)	882/886 (99%)	975/976 (99%)	974/977 (99%)	961/963 (99%)	965/966 (99%)
Accession no.	AJ705037.1	AJ705037.1	FJ169659.1	FJ169659.1	AJ745682.1	AJ745682.1
3	*B. ficariarum*	*B. ficariarum*	*B. squamosa*	*B. squamosa*	*B. squamosa*	*B. squamosa*
Identities	880/884 (99%)	882/886 (99%)	975/976 (99%)	974/977 (99%)	960/963 (99%)	964/966 (99%)
Accession no.	AJ705016.1	AJ705016.1	AJ716098.1	AJ716098.1	FJ169682.1	FJ169682.1
4	*B. elliptica*	*B. elliptica*	*B. elliptica*	*B. elliptica*	*B. squamosa*	*B. squamosa*
Identities	880/884 (99%)	882/886 (99%)	975/976 (99%)	974/977 (99%)	960/963 (99%)	964/966 (99%)
Accession no.	AJ705010.1	AJ705010.1	AJ716071.1	AJ716071.1	AJ745707.1	AJ745707.1
5	*B. squamosa*	*B. squamosa*	*B. elliptica*	*B. elliptica*	*B. elliptica*	*B. elliptica*
Identities	879/884 (99%)	881/886 (99%)	974/976 (99%)	973/977 (99%)	958/963 (99%)	962/966 (99%)
Accession no.	EU519214.1	EU519214.1	AM232669.1	AM232669.1	EU514477.1	EU514477.1

## Mycological features of isolated *Botrytis* species

The light microscopy findings stained with lactophenol cotton-blue showed that the mycelium was branched with septa and the conidiophores were also septated. The conidia were single-celled with egg-shaped hyaline and the diameter ranged from approximately 2–3 × 5–8 μm (Fig. 3b). A growth temperature test was performed with incubation on PDA for 7 days at 25 °C and 35 °C. The growth of the colonies was observed at 25 °C but not at 35 °C.

Multilocus gene sequence analyses targeting the internal transcribed spacer (ITS) 1 and 2 region and domain 1 and 2 (D1/D2) region of 28S rRNA were performed for species identification. The genomic DNA of this isolate was prepared using Go Taq® Green Master Mix (Promega Corporation, WI, USA), and PCR was carried out in a Veriti® 96-Well Thermal Cycler (Thermo Fisher Scientific K.K., Yokohama, Japan). The PCR was performed using the primer pair ITS1 and ITS4 for the ITS region, and the primer pair NL-1 and NL-4 for the D1/D2 region (Table 2) [5, 6]. Sanger sequencing data of the PCR products were compared with those of the GenBank database search using BLAST. The isolate had 99% similarity to sequences from *B. cinerea*, its teleomorph (*Botryotinia fuckeliana*), *B. fabiopsis* and *B. elliptica* at the ITS region, and *B. cinerea* at the D1/D2 region (see Additional file 1: Figures S1 and S2, and Table 3).

As the sequences of ITS and D1/D2 regions do not permit sufficient resolution to the species level in the genus *Botrytis* [7], we further analyzed three single-copy nuclear DNA genes encoding glyceraldehyde-3-phosphate dehydrogenase (*G3PDH*), heat-shock protein 60 (*HSP60*), and DNA-dependent RNA polymerase subunit II (*RPB2*), which are known to be more discriminatory in attaining the *Botrytis* species level. The PCR was performed using the primer pairs G3PDHfor/G3PDHrev, HSP60for/HSP60rev and RPB2for/RPB2rev for *G3PDH*, *HSP60* and *RPB2*, respectively (Table 2) [7]. The PCR products were sequenced and the result of the GenBank database search using BLAST showed that the isolate was most similar to *B. elliptica* (see Additional file 1: Figures S3-S5 and Table 4).

The antifungal susceptibility test of the isolated *Botrytis* species was performed using the broth microdilution assay according to the Clinical and Laboratory Standards Institute approved standard M38-A2 guideline for molds. We tried to evaluate the minimal inhibitory concentration (MIC) of antifungal agents including amphotericin B, micafungin, voriconazole, fluconazole, itraconazole, miconazole, and flucytosine. No growth was observed at any MIC for any antifungal agents including the control media.

## Discussion and conclusions

This case is the first report of an apparently immunocompetent patient with pulmonary *Botrytis* sp. infection, which has not recurred after lung resection. Furthermore, the patient did not require any additional medication.

*Botrytis* species are well known fungal pathogens of various plants and agricultural products but little is known about their pathogenicity in humans. Two clinical studies reported an association between asthma and positive skin prick test reaction to molds including *B. cinerea* [8, 9]. Korhonen [8] reported that the most common responsive molds were *B. cinerea*, *Aspergillus fumigatus* and *Cladosporium herbarum* in young Finnish children newly diagnosed with asthma. Immonen [9] found that allergy toward *B. cinerea* is just as prevalent as allergy toward *A. fumigatus*, *Alternaria alternata*, and *C. herbarum* in Finnish school children having asthma or in those suspected of asthma. Specific IgE antibodies to molds were investigated using a standard mold test panel (Phadebas RAST®) and an extended mold panel, in which *B. cinerea* was included, in two studies [10, 11]. Karlsson-Borgå [10] reported that *B. cinerea* was the second most prevalent mold allergen in Sweden and Denmark, and the most prevalent in the USA in patients with suspected mold allergy. Koivikko [11] found that *B. cinerea* was the fourth most prevalent fungal allergy in 121 asthmatic children. Similar to other fungi, *B. cinerea* contains 1, 3-β-D-glucan and chitin in its cell wall. Studies have shown that inhalation of this particular 1, 3-β-D-glucan can elicit respiratory inflammation and chitin might also be involved in allergic reactions upon frequent exposure to this polysaccharide [3].

Cases of hypersensitivity pneumonitis/allergic alveolitis caused by *B. cinerea* have been reported in two farm workers working with noble rot grapes (wine grower’s lung) [12]. They inhaled spores of *B. cinerea* during grape gathering. Their chest radiograph showed a reticular shadow indicative of pulmonary fibrosis. *B. cinerea* specific IgG antibody was identified using the Ouchterlony immunodiffusion test and the immunofluorescence test in both patients.

There have been no reports of *Botrytis* infection in humans even though many people likely inhale spores of *Botrytis* species. Detection of saprophytic molds from sputum or bronchoalveolar lavage samples has generally been considered as contamination or colonization [13]. In our case *Botrytis* was detected in pure culture from a resected lung nodule. Fungal infections occur in patients with risk factors such as environmental factors, primary or acquired immunodeficiency, and structural lung diseases. Our patient had not stayed or worked in an environment where significant exposure to spores of *Botrytis* species was likely. He had diabetes mellitus, which is a known risk factor for fungal infections [14], but the patient’s diabetes was strictly controlled and hemoglobin A1c was normal. He did not have diseases causing immunosuppression such as acquired immunodeficiency syndrome or hematological malignancy, and took neither corticosteroids, nor cytotoxic agents. Recently, biological defense mechanisms against fungal infections have been elucidated [15] and primary immunodeficiencies including chronic granulomatous disease (CGD) or caspase recruitment domain family member 9 deficiency can also predispose to invasive fungal diseases [16]. Although we did not test for CGD or other genetic markers that may confer susceptibility, he had never experienced any fungal infections until this presentation and his family also had no fungal infections. Pulmonary aspergillosis that is unrelated to immunosuppression requires previous airway damage, such as bronchiectasis or bullous disease. This patient had a history of 60 pack-years of smoking cigarettes. The fungus ball can be formed by proliferation of fungi saprophytically in emphysematous bulla. This could not be demonstrated because he had not received a CT scan prior to his clinical presentation within our hospital.

Alternatively, *Botrytis* lung infection may have been misdiagnosed as *Aspergillus* infection, when we found Y-shaped filamentous fungi with septa in the resected lung tissue histopathologically without microbiological examination.

The antifungal susceptibility test of the isolated *Botrytis* species was performed using the Clinical and Laboratory Standards Institute M38-A2 broth microdilution method. No growth was observed at any MIC for any of the antifungal agents including the control media. We tried to incubate the isolate in liquid media including Roswell Park Memorial Institute media 1640 and Sabouraud liquid broth media, but no growth was observed (data not shown). This suggests that the isolated *Botrytis* species may be difficult to incubate in liquid media.

In conclusion, we report the first case of an apparently immunocompetent patient with pulmonary *Botrytis* sp. infection, which has not recurred after lung resection without any additional medication. Precise evaluation is necessary for the diagnosis of pulmonary *Botrytis* infection, because it is indistinguishable from other filamentous fungi both radiologically and by histopathology. Furthermore, the etiology and pathophysiology of pulmonary *Botrytis* infection remains unclear. Further accumulation and analysis of *Botrytis* cases is warranted.

## Additional file

Additional file 1: **Figure S1.** Sequence data of internal transcribed spacer (ITS) region. **Figure S2.** Sequence data of domain 1 and 2 region. **Figure S3.** Sequence data of glyceraldehyde-3-phosphate dehydrogenase (*G3PDH*) gene. **Figure S4.** Sequence data of heat-shock protein 60 (*HSP60*) gene. **Figure S5.** Sequence data of DNA-dependent RNA polymerase subunit II (*RPB2*) gene. (PDF 383 kb)

## Data Availability

All data generated or analyzed during this study are included in this published article and its supplementary information files.

## Abbreviations

BLAST: Basic Local Alignment Search Tool
CGD: Chronic granulomatous disease
CT: Computed tomography
D1/D2: Domain 1 and 2
*G3PDH*: Glyceraldehyde-3-phosphate dehydrogenase
*HSP60*: Heat-shock protein 60
ITS: Internal transcribed spacer
MIC: Minimal inhibitory concentration
PCR: Polymerase Chain Reaction
PDA: Potato dextrose agar
*RPB2*: DNA-dependent RNA polymerase subunit II
rRNA: ribosomal ribonucleic acid
