# Supplementary material for: A case report of pulmonary Botrytis sp. infection in an apparently healthy individual
Source: BMC Infect Dis. 2019 Aug 2;19:684. doi: 10.1186/s12879-019-4319-2 (PMC6679495; doi:10.1186/s12879-019-4319-2)
Supplement: Supplementary file 1 — Figure S1. Sequence data of internal transcribed spacer (ITS) region. Figure S2. Sequence data of domain 1 and 2 region. Figure S3. Sequence data of glyceraldehyde-3-phosphate dehydrogenase (G3PDH) gene. Figure S4. Sequence data of heat-shock protein 60 (HSP60) gene. Figure S5. Sequence data of DNA-dependent RNA polymerase subunit II (RPB2) gene. (PDF 383 kb) [file 12879_2019_4319_MOESM1_ESM.pdf]

Title: A case report of pulmonary *Botrytis* sp. infection in an apparently healthy individual

Journal: BMC Infectious Diseases

Authors:

Seishu Hashimoto, MD, Department of Respiratory Medicine, Tenri Hospital, Tenri, Japan

Eisaku Tanaka, PhD, Department of Respiratory Medicine, Tenri Hospital, Tenri, Japan

Masakuni Ueyama, MD, Department of Respiratory Medicine, Tenri Hospital, Tenri, Japan

Satoru Terada, MD, Department of Respiratory Medicine, Tenri Hospital, Tenri, Japan

Takashi Inao, MD, Department of Respiratory Medicine, Tenri Hospital, Tenri, Japan

Yusuke Kaji, MD, Department of Respiratory Medicine, Tenri Hospital, Tenri, Japan

Takehiro Yasuda, MD, Department of Respiratory Medicine, Tenri Hospital, Tenri, Japan

Takashi Hajiro, PhD, Department of Respiratory Medicine, Tenri Hospital, Tenri, Japan

Tatsuo Nakagawa, PhD, Department of Thoracic Surgery, Tenri Hospital, Tenri, Japan

Satoshi Noma, PhD, Department of Radiology, Tenri Hospital, Tenri, Japan

Gen Honjo, MD, Department of Pathology, Tenri Hospital, Tenri, Japan

Yoichiro Kobashi, MD, Department of Pathology, Tenri Hospital, Tenri, Japan

Noriyuki Abe, Department of Clinical Laboratory, Tenri Hospital, Tenri, Japan

Katsuhiko Kamei, PhD, Division of Clinical Research, Medical Mycology Research Center, Chiba University, Chiba, Japan

Yoshio Taguchi, MD, Department of Respiratory Medicine, Tenri Hospital, Tenri, Japan

Corresponding author: Seishu Hashimoto, MD

Department of Respiratory Medicine, Tenri Hospital, Tenri, Japan

E-mail: hassy@tenriyorozu.jp

Additional file 1: Figure S1 Sequence data of internal transcribed spacer (ITS) region.

ITS forward
TGCCNGAAGGGTAGACCTCCCACCCTTGTGTATTATTACTTTGTTGCTTTGGCGAGCTGCCTTCGGG
CCTTGTATGCTCGCCAGAGAAAACCAAAACTCTTTTTATTAATGTCGTCTGAGTACTATATAATAG
TTAAAACTTTCAACAACGGATCTCTTGGTTCTGGCATCGATGAAGAACGCAGCGAAATGCGATAA
GTAATGTGAATTGCAGAATTCAGTGAATCATCGAATCTTTGAACGCACATTGCGCCCCTTGGTATT
CCGGGGGGCATGCCTGTTCGAGCGTCATTTCAACCCTCAAGCTTAGCTTGGTATTGAGTCTATGTC
AGTAATGGCAGGCTCTAAAATCAGTGGCGGCGCCGCTGGGTCCTGAACGTAGTAATATCTCTCGTT
ACAGGTTCTCGGTGTGCTTCTGCCAAAACCCAAATTTTTCTATGGTTGACCTCGGATCAGGTAGGG
ATACCCGCTGAACTTAAGCATATCAATAG

ITS reverse
GATCCGAGGTCACCATAGAAAAATTTGGGTTTTGGCAGAAGCACACCGAGAACCTGTAACGAGAG
ATATTACTACGTTCAGGACCCAGCGGCGCCGCCACTGATTTTAGAGCCTGCCATTACTGACATAGA
CTCAATACCAAGCTAAGCTTGAGGGTTGAAATGACGCTCGAACAGGCATGCCCCCCGGAATACCA
AGGGGCGCAATGTGCGTTCAAAGATTCGATGATTCACTGAATTCTGCAATTCACATTACTTATCGC
ATTTCGCTGCGTTCTTCATCGATGCCAGAACCAAGAGATCCGTTGTTGAAAGTTTTAACTATTATAT
AGTACTCAGACGACATTAATAAAAAGAGTTTTGGTTTTCTCTGGCGAGCATACAAGGCCCGAAGG
CAGCTCGCCAAAGCAACAAAGTAATAATACACAAGGGTGGGAGGTCTACCCTTTCGGGCATGAAC
TCTGTAATGATCCTTCCGCAGGTT

Figure S2 Sequence data of domain 1 and 2 region.

Domain 1 and 2 forward
CAGTACGGCGAGTGAAGCGGTAAAAGCTCAAATTTGAAATCTGGCTCTTTTAGAGTCCGAATTGTA
ATTTGTAGAAGATGCTTCGGGTGTGGTTCCGGTCTAAGTTCCTTGGAACAGGACGTCATAGAGGGT
GAGAATCCCGTATGTGACTGGATACCTATGCTCATGTGAAGCTCTTTCGACGAGTCGAGTTGTTTG
GGAATGCAGCTCAAAATGGGAGGTATATTTCTTCTAAAGCTAAATATTGGCCAGAGACCGATAGC
GCACAAGTAGAGTGATCGAAAGATGAAAAGCACTTTGGAAAGAGAGTTAAACAGTACGTGAAAT
TGTTGAAAGGGAAGCGCTTGCAATCAGACTTGCACTTGGTGTTCATCAGGGTCTCGTACCCTGTGT
ACTTCATCAAGTTCAGGCCAGCATCAGTTTGAGTGGTTAGATAAAGGCTTAGAGAATGTGGCCCTC
TTCGGGGGGTGTTATAGCTCTAGGTGCAATGTAGCCTACTTGGACTGAGGACCGCGCTTCGGCTAG
GATGCTGGCGTAATGGTTGTAAGCGACCCGTCTTNACAC

Domain 1 and 2 reverse
GCATCCTAGCCGAGCGCGGTCCTCAGTCCAAGTAGGCTACATTGCACCTAGAGCTATAACACCCCC
CGAAGAGGGCCACATTCTCTAAGCCTTTATCTAACCACTCAAACTGATGCTGGCCTGAACTTGATG
AAGTACACAGGGTACGAGACCCTGATGAACACCAAGTGCAAGTCTGATTGCAAGCGCTTCCCTTT
CAACAATTTCACGTACTGTTTAACTCTCTTTCCAAAGTGCTTTTCATCTTTCGATCACTCTACTTGTG
CGCTATCGGTCTCTGGCCAATATTTAGCTTTAGAAGAAATATACCTCCCATTTTGAGCTGCATTCCC
AAACAACTCGACTCGTCGAAAGAGCTTCACATGAGCATAGGTATCCAGTCACATACGGGATTCTC
ACCCTCTATGACGTCCTGTTCCAAGGAACTTAGACCGGAACCACACCCGAAGCATCTTCTACAAAT
TACAATTCGGACTCTAAAAGAGCCAGATTTCAAATTTGAGCTTTTACCGCTTCACTCGCCGTTACT
GAGGTAATCCCTGTTGGTTTCTTTTCCTC

Figure S3 Sequence data of glyceraldehyde-3-phosphate dehydrogenase (*G3PDH*) gene.

***G3PDH* forward**

ATTAGTTTCCGCTATCGGACCTCCCGCAGATTTCAAGGACCCGAGCTAATCTATTTTATGTACAGG
CATACATGTTGAAGTATGATTCCACCCACGGTCAATTCAAGGGTGATATCAAGGTCCTTTCCGATG
GATTGGAGGTCAATGGCAAGAAGGTCAAGTTCTACACCGAAAGAGACCCAGCCAACATCCCATGG
GCTGAGTCTGAGGCATACTACGTTGTCGAGTCCACCGGTGTTTTCACCACCACCGAGAAGGCTAAG
GCCCATTTGAAGGGTGGTGCCAAGAAGGTTGTTATCTCTGCTCCTTCTGCCGATGCCCCAATGTAC
GTTATGGGTGTCAACAACGAGACCTACAAGGGCGATGTTGATGTTATCTCCAACGCCTCTTGCACA
ACCAACTGCTTGGCTCCTCTCGCCAAGGTCATCAACGATGAGTTCACCATCATCGAAGGTTTGATG
ACCACCATCCACTCCTACACCGCCACCCAAAAGACCGTCGATGGTCCATCCGCTAAGGATTGGCGT
GGAGGACGTACCGCTGCTCAAAACATCATCCCATCGAGCACCGGTGCTGCCAAGGCCGTCGGAAA
GGTTATCCCAGAGCTTAACGGCAAACTCACCGGAATGTCCATGCGTGTTCCAACTGCCAACGTCTC
GGTTGTTGACTTGACTGTCCGCATTGAGAAGGGTGCTTCTTATGATGAGATCAAGGCCGTCATCAA
GAAGGCTGCTGATGGTCCTCTCAAGGGTGAGTTACTCTATTAATATTTCTTCCGTTTTAATTTACTA
ATCGTAATACAGGCATATTGGCTTACACTGAGGACGATGTTGTCTCCACTGACATGAACGGTGACA
ACCACTCCTCCATCTTCGATGCCAAGGCCGGTATCTCCCTCAACGCAAACTTCGTCAAGTTGGTTTC
CTGGTAC

***G3PDH* reverse**

GGGAGATACCGGCCTTGGCATCGAAGATGGAGGAGTGGTTGTCACCGTTCATGTCAGTGGAGACA
ACATCGTCCTCAGTGTAAGCCAATATGCCTGTATTACGATTAGTAAATTAAAACGGAAGAAATATT
AATAGAGTAACTCACCCTTGAGAGGACCATCAGCAGCCTTCTTGATGACGGCCTTGATCTCATCAT
AAGAAGCACCCTTCTCAATGCGGACAGTCAAGTCAACAACCGAGACGTTGGCAGTTGGAACACGC
ATGGACATTCCGGTGAGTTTGCCGTTAAGCTCTGGGATAACCTTTCCGACGGCCTTGGCAGCACCG
GTGCTCGATGGGATGATGTTTTGAGCAGCGGTACGTCCTCCACGCCAATCCTTAGCGGATGGACCA
TCGACGGTCTTTTGGGTGGCGGTGTAGGAGTGGATGGTGGTCATCAAACCTTCGATGATGGTGAAC
TCATCGTTGATGACCTTGGCGAGAGGAGCCAAGCAGTTGGTTGTGCAAGAGGCGTTGGAGATAAC
ATCAACATCGCCCTTGTAGGTCTCGTTGTTGACACCCATAACGTACATTGGGGCATCGGCAGAAGG
AGCAGAGATAACAACCTTCTTGGCACCACCCTTCAAATGGGCCTTAGCCTTCTCGGTGGTGGTGAA
AACACCGGTGGACTCGACAACGTAGTATGCCTCAGACTCAGCCCATGGGATGTTGGCTGGGTCTCT
TTCGGTGTAGAACTTGACCTTCTTGCCATTGACCTCCAATCCATCGGAAAGGACCTTGATATCACC
CTTGAATTGACCGTGGGTGGAATCATACTTCAACATGTATGCCTGTACATAAAATAGATTAGCTCG
GGTCCTTGAAATCTGCGGGAGGTCCGATAGCGGAAACTTACAGCATATTCAGTCTCGATAAAAGG
GTCGTTGACAGCG

Figure S4 Sequence data of heat-shock protein 60 (*HSP60) gene.*

***HSP60* forward**

GATATGACATACATATCGGGTGATTGTAAACTAATCAAGTTTTTGATTTAGGAGCTCAAATTCGGT
GTTGAGGGCAGAGCAGCTCTTCTTGCTGGTGTTGAGACTTTGGCGAAAGCTGTTGCCACAACCTTA
GGTCCCAAAGGCCGAAATGTTCTTATTGAGTCAGCATATGGCTCCCCAAAGATCACTAAAGGTTTG
CGAACTCCTCGGTTACCTAGTTGTAAAATTCTAATCGTTGGTGAATAGATGGTGTAACTGTTGCCA
GAGCTATTTCCCTCAAGGACAAGTTCGAGAACCTCGGTGCTAGACTCATCCAAGATGTTGCCTCGA
AAACCAACGAGACCGCTGGTGATGGAACCACAACTGCTACTGTCCTTGCTAAATCTATCTTCTCCG
AGACCGTAAAGAACGTCGCCGCAGGATGCAACCCAATGGACTTGCGCAGAGGAACCCAAGCCGC
CGTGGAGGCCGTTGTTGAGTTTTTGCAAAAGAACAAGCGTGATATCACAACCAGCGAGGAAATCG
CACAAGTTGCGACTATCAGTGCAAACGGTGATACCCACATCGGAAAGTTGATTGCCAACGCTATG
GAGAAGGTTGGAAAGGAAGGTGTCATCACAGTCAAGGAGGGAAAGACCATGGAGGATGAACTCG
ACATTACCGAGGGAATGAGATTTGACCGCGGTTATGTCTCCCCATACTTCATCACCGATACCAAGT
CGCAAAAGGTCGAATTCGAGAAGCCATTGATCCTCCTTTCTGAGAAGAAGATCTCAAACGTCCAA
GATATTATCCCAGCACTTGAGGCATCTACTCAACTTCGCCGTCCTTTGGTCATCATTGCTGAAGATA
TCGATGGAGAAGCTCTCGCTGTATGCATTCTCAACAAGCTCCGTGGTCAACTCCAAGTTGCCGCTG
TCAAGGCCCCCGGTTTCGGTGATAACCGAAAGTCTATCCTCGGCGATCTCGGTATCCTGACCAATG
CTACCGTCTTCACTGACGAGCTTGA

***HSP60* reverse**

AGTCTCANNTNNNNNNNGTCAGTGAAGACGGTAGCATTGGTCAGGATACCGAGATCGCCGAGGAT
AGACTTTCGGTTATCACCGAAACCGGGGGCCTTGACAGCGGCAACTTGGAGTTGACCACGGAGCT
TGTTGAGAATGCATACAGCGAGAGCTTCTCCATCGATATCTTCAGCAATGATGACCAAAGGACGG
CGAAGTTGAGTAGATGCCTCAAGTGCTGGGATAATATCTTGGACGTTTGAGATCTTCTTCTCAGAA
AGGAGGATCAATGGCTTCTCGAATTCGACCTTTTGCGACTTGGTATCGGTGATGAAGTATGGGGAG
ACATAACCGCGGTCAAATCTCATTCCCTCGGTAATGTCGAGTTCATCCTCCATGGTCTTTCCCTCCT
TGACTGTGATGACACCTTCCTTTCCAACCTTCTCCATAGCGTTGGCAATCAACTTTCCGATGTGGGT
ATCACCGTTTGCACTGATAGTCGCAACTTGTGCGATTTCCTCGCTGGTTGTGATATCACGCTTGTTC
TTTTGCAAAAACTCAACAACGGCCTCCACGGCGGCTTGGGTTCCTCTGCGCAAGTCCATTGGGTTG
CATCCTGCGGCGACGTTCTTTACGGTCTCGGAGAAGATAGATTTAGCAAGGACAGTAGCAGTTGTG
GTTCCATCACCAGCGGTCTCGTTGGTTTTCGAGGCAACATCTTGGATGAGTCTAGCACCGAGGTTC
TCGAACTTGTCCTTGAGGGAAATAGCTCTGGCAACAGTTACACCATCTATTCACCAACGATTAGAA
TTTTACAACTAGGTAACCGAGGAGTTCGCAAACCTTTAGTGATCTTTGGGGAGCCATATGCTGACT
CAATAAGAACATTTCGGCCTTTGGGACCTNAAGGTTGTGGCAACAGCTTTCGCCAAAGTCTCAACA
CCAGCAAGAAGAGCTGCTCTGCCCTCAACACCGAATTTGAGCTCCTAAATCAAAAACTTGATTAGT
TTACNATCACCCGATATGTATGTCATATCTCAGAGCGACATGNACTGAAGTTAACC

Figure S5 Sequence data of DNA-dependent RNA polymerase subunit II (*RPB2*) gene.

***RPB2* forward**
TTTACTTGCAAGTTGTTCAGAAATCTGTTCCGTAGATTGACAACGGATGTGTACAGATACTTGCAA
CGCTGCGTGGAAAACAACCGAGAGTTTAATTTAACTTTGGGTGTGAAATCCACAACAATCACGAA
CGGTCTGAAATATTCTTTGGCCACAGGTAACTGGGGTGACCAGAAGAAGGCAGCAAGTTCTACCG
CTGGTGTGTCTCAAGTGTTGAACAGATATACTTTTGCTTCAACGCTTTCTCATTTGCGCCGAACCAA
TACACCTATCGGACGTGATGGAAAGATCGCCAAACCTAGACAACTGCATAATACTCATTGGGGCT
TGGTCTGTCCTGCAGAGACGCCCGAAGGTCAAGCTTGTGGTTTGGTTAAGAATTTGGCTTTGATGT
GTTACGTTACAGTTGGTACGCCAAGTGATCCAATCGTCGAGTTCATGATTCAAAGAAACATGGAA
GTGTTGGAGGAGTACGAACCACTCCGAGCACCCAATGCAACAAAGGTTTTCGTCAATGGTGTTTG
GGTTGGTATTCATCGAGATCCTGCTCATTTGGTCAAATGTGTCCAAGATCTTCGTAGATCACACTTG
ATCTCTCATGAAGTTTCACTTATTCGGGAAATTCGTGACAGAGAGTTCAAGATTTTCACCGATGCA
GGACGAGTGTGCAGACCTTTATTGGTTATTGACAATGATCCTGACAGCACCAACAAAGGTAACTTG
GTATTGAATAAGGACCACATTCACCGTCTGGAGGAAGATCAGACGATGCCAGCCAACATGGATAA
GGGTGATAAACTAAGGGAAGGATACTATGGATTCCAAGGTTTGATTAATGATGGTGTGGTTGAGT
ATCTGGACGCCGAGGAAGAAGAGACCGTCATGATTACCATGACACCTGAAGATCTGGACATCTCT
CGACAACTTCAGGCTGGTTATCAAATTCGTCCTGATGAAAGTGGTGATTTGACAAGCGTGTCAAGG
CA

***RPB2* reverse**
TTTGTAGCAATAGAATTAGCCTTGAATGAATAAATTCATTGAACTTAAAGCTTACCTGATTGTGAT
CCGGGAAGGGAATAATGCTTGCGCAGATGCCCAAGATCATACTTGGATGAATTTCACAATGAGTC
CAGACATGGGCAGTTGGATTGATAGGTGCCTTGACACGCTTGTTCAAATCACCACTTTCATCAGGA
CGAATTTGATAACCAGCCTGAAGTTGTCGAGAGATGTCCAGATCTTCAGGTGTCATGGTAATCATG
ACGGTCTCTTCTTCCTCGGCGTCCAGATACTCAACCACACCATCATTAATCAAACCTTGGAATCCA
TAGTATCCTTCCCTTAGTTTATCACCCTTATCCATGTTGGCTGGCATCGTCTGATCTTCCTCCAGAC
GGTGAATGTGGTCCTTATTCAATACCAAGTTACCTTTGTTGGTGCTGTCAGGATCATTGTCAATAAC
CAATAAAGGTCTGCACACTCGTCCTGCATCGGTGAAAATCTTGAACTCTCTGTCACGAATTTCCCG
AATAAGTGAAACTTCATGAGAGATCAAGTGTGATCTACGAAGATCTTGGACACATTTGACCAAAT
GAGCAGGATCTCGATGAATACCAACCCAAACACCATTGACGAAAACCTTTGTTGCATTGGGTGCTC
GGAGTGGTTCGTACTCCTCCAACACTTCCATGTTTCTTTGAATCATGAACTCGACGATTGGATCACT
TGGCGTACCAACTGTAACGTAACACATCAAAGCCAAATTCTTAACCAAACCACAAGCTTGACCTTC
GGGCGTCTCTGCAGGACAGACCAAGCCCCAATGAGTATTATGCAGTTGTCTAGGTTTGGCGATCTT
TCCATCACGTCCGATAGGTGTATTGGTTCGGCGCAAATGAGAAAGCGTTGAAGCAAAAGTATATC
TGTTCAACACTTGAGACACACCAGCGGTAGAACTTGCTGCCTTCTTCTGGTCACCCCAGTTACCTG
TGG
